# A Culturally Adapted Timed-Activity RCT for Latinos With ADRD and Caregivers: Feasibility, Acceptability, and Effects

**DOI:** 10.1093/geroni/igab046.2092

**Published:** 2021-12-17

**Authors:** G Adriana Perez, Liming Huang

**Affiliations:** 1 University of Pennsylvania School of Nursing, Phildelphia, Pennsylvania, United States; 2 University of Pennsylvania, Philadelphia, Pennsylvania, United States

## Abstract

Latinos are twice as likely to develop Alzheimer’s disease (AD) compared to non-Latino whites, yet, account for <2% of clinical trial participants in AD research. This randomized controlled trial examined the feasibility, acceptability and effects of a culturally-adapted timed-activity intervention designed to promote quality of life (QOL) and reduce behavioral symptoms in older Latinos with AD and their caregivers. Healthy Patterns [Pautas Saludables] was implemented among 40 Spanish-speaking dyads. Measures assessed at baseline and 4 weeks post-intervention, indicate improvements in sleep efficiency (p=.06) and QOL (p=.01) among intervention participants. Pautas Saludables was found to be feasible and acceptable. Intervention attendance rate was >90% with low attrition (n=0); no adverse events. Most (74%) rated timed-activity sessions as helpful and appropriate; 58% recommended refreshers. Results provide evidence that Latinos with AD will participate in clinical trials and can improve on key health outcomes, when interventions are adapted to meet their cultural needs.

